# Intra-Articular Injection of Parathyroid Hormone (1–34) Enhances Meniscal Healing at the Site of a Radial Tear in the Rabbit Medial Meniscus

**DOI:** 10.3390/ijms27146215

**Published:** 2026-07-12

**Authors:** Shuko Tsumoto, Yusuke Hashimoto, Ken Iida, Kazuya Nishino, Hidetomi Terai

**Affiliations:** 1 Department of Orthopedic Surgery, Graduate School of Medicine, Osaka Metropolitan University, Osaka 545-8585, Japan; duponddubont@gmail.com (S.T.); e_ken_e_kodayo@yahoo.co.jp (K.I.); kazuya_best@hotmail.com (K.N.);; 2 Department of Sport Sciences, Graduate School of Sport Sciences, Osaka University of Health and Sport Sciences, Sennan 590-0495, Japan

**Keywords:** parathyroid hormone, meniscal healing, medial meniscal radial tear

## Abstract

In this study, we assessed the efficacy of a single intra-articular injection of parathyroid hormone (PTH) (1–34) to enhance the meniscal healing of a medial meniscal radial tear. A radial tear involving two-thirds of the meniscal width was created in the anterior medial meniscus of the bilateral knees in 30 Japanese white rabbits that were injected with either PTH (1–34) or phosphate-buffered saline. Macroscopic, biomechanical, and histological assessments of the meniscus were performed at 4, 8, and 12 weeks post-surgery. PTH injection resulted in an improved meniscal healing rate at 4 and 8 weeks compared with phosphate-buffered saline (*p* = 0.05 and *p* < 0.05, respectively). After 4 weeks, PTH injection led to a higher circumferential strength in biomechanical testing. The meniscal healing score exhibited a trend toward improvement, reaching borderline statistical significance at 4 weeks with PTH injection (1.6 ± 1.3) than with phosphate-buffered saline (0.5 ± 0.8) (*p* = 0.05). In the outer two-thirds of the meniscal width, picrosirius red staining revealed greater type I collagen content following PTH injection. A single injection of PTH (1–34) into the knee joint improved meniscal healing in a rabbit medial meniscus radial tear model at 4 and 8 weeks, which was accompanied by increased type I collagen content around the healing site.

## 1. Introduction

A meniscal tear is a common injury of the knee joint and is present in various injury patterns, including horizontal, longitudinal, radial, and root tears [1]. Owing to the limited vascularity of the tissue, most meniscal tears do not heal spontaneously, and surgical intervention is indicated based on the symptoms and injury pattern [2]. Among the different types, radial tears represent the most challenging injury pattern [1,3]. In recent years, radial tears have been repaired using various repair techniques and devices [1,4]. However, the clinical outcomes achieved using these techniques and devices have remained limited [5].

To enhance the healing of meniscal tears, recent advances in tissue engineering and regenerative medicine have focused on the use of cells, scaffolds, and bioactive molecules, either independently or in combination. Mesenchymal stem cells (MSCs) are widely used in cell-based therapies for meniscal repair and regeneration [6,7]. The application of MSCs has shown favorable results, although some challenges remain, such as patients requiring multiple operations [6] and the lack of a clearly established optimal MSC source for treating meniscal tears [8].

Parathyroid hormone (PTH) (1–34) is widely used in the clinical setting as an anabolic therapy for osteoporosis. In osteoarthritis treatment studies, PTH (1–34) is used to inhibit hypertrophy of bone marrow MSCs (BMSCs) during chondrogenic differentiation [9]. More recently, PTH (1–34) has been used in in vivo studies to repair meniscal defects. In a rat model of meniscal defects, transplanted Achilles tendons treated with PTH (1–34) promoted meniscal regeneration [10]. In a canine model, combining BMSCs and PTH (1–34) promoted the regeneration of a three-dimensional (3D)-printed meniscal scaffold [9]. The effect of PTH (1–34) on meniscal defects has been widely investigated; nonetheless, limited evidence exists regarding its effect on radial tears of the medial meniscus.

The aim of this study was to assess the efficacy of a single intra-articular injection of PTH (1–34) in enhancing meniscal healing of medial meniscal radial tears in a rabbit model. We hypothesized that intra-articular injection of PTH (1–34) would enhance meniscal healing and result in an improved healing score and strength compared to phosphate-buffered saline (PBS) injection.

## 2. Results

No complications or unexpected adverse events such as infection, wound breakdown, or unexpected death were observed after surgery. Therefore, no rabbit was excluded from this study. The interobserver reliability of the histologic score was 0.942 (95% confidence interval [CI], 0.787–0.985; *p* < 0.001). Furthermore, the intraobserver reliability of the histologic score was excellent, with an interclass correlation coefficient of 0.992 (95% CI, 0.979–0.997; *p* < 0.001).

### 2.1. Macroscopic Evaluation and Biomechanical Testing

At 4 weeks, 5 of 10 menisci were healed in the PTH group compared with 1 of 10 in the control group (*p =* 0.05). At 8 weeks, healing was observed in 3 of the 10 menisci in the PTH group but in none in the control group (*p* = 0.01). At 12 weeks, 2 of the 10 menisci healed in both groups (*p* = 1.0). None of the specimens showed healing extending to the inner third of the meniscus.

**Figure 1:**
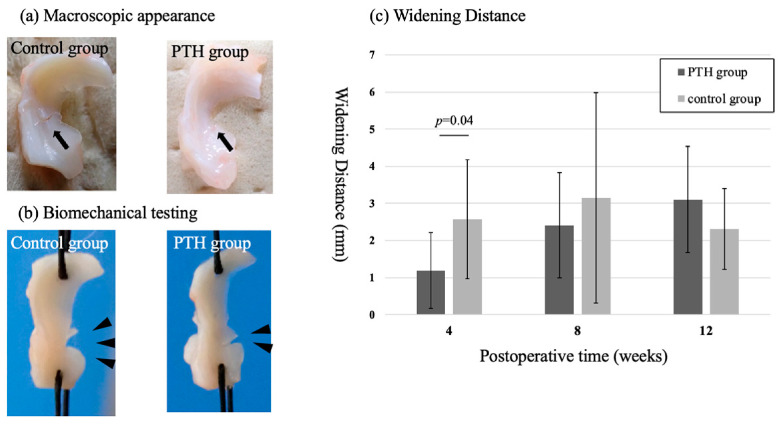
Representative images of macroscopic appearance and biomechanical testing of the medial meniscus at 4 weeks after surgery in the control and parathyroid hormone (PTH) groups and changes in widening distance. The radial tear is indicated by a black arrow. The widening distance is indicated by an arrowhead. (**a**) Representative macroscopic appearance of the control (**left**) and PTH (**right**) groups. (Left knee): The meniscus remained unhealed in the control group, whereas healing was observed in the PTH group, with tears remaining only in the white zone. (**b**) Representative biomechanical testing of the control (**left**) and PTH (**right**) groups. (Left knee): A substantial gap was observed in the control under tensile loading, whereas the gap remained relatively small in the PTH group. (**c**) Changes in widening distance. The widening distance was significantly smaller in the PTH group than in the control group at 4 weeks postoperatively (*p* = 0.04). Data are presented as mean ± SD. *n* = 10 per group at each time point.

The widening distance at 4 weeks was 1.19 ± 1.02 mm in the PTH group compared with 2.63 ± 1.71 mm in the control group (*p* = 0.04). At 8 weeks, the widening distance was 2.41 ± 1.42 mm in the PTH group and 3.15 ± 2.84 mm in the control group (*p* = 0.79). At 12 weeks, it was 3.11 ± 1.43 mm and 2.31 ± 1.09 mm in the PTH and control groups, respectively (*p* = 0.32) (Figure 1).

### 2.2. Histologic Evaluation

**Figure 2:**
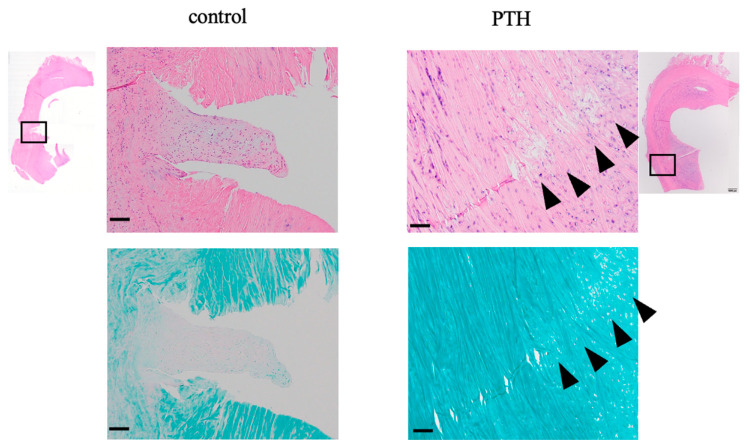
Histologic findings of the meniscus of control and parathyroid hormone (PTH) groups 4 weeks postoperatively. The axial section of the tear site was stained with hematoxylin–eosin and safranin O. In the PTH group, the bond that is observed at the site of meniscus healing is depicted by an arrowhead. In the control group, scar tissue is observed between the gaps, although no bridge connects the gaps. Hence, it represents a meniscal healing score (MHS) of 1. In the PTH group, a connection is present between the tear sites, representing an MHS of 3. Scale bar = 100 μm. *n* = 10 per group at each time point.

Histologically, a connected bond was observed in the healed meniscus, and scar tissue or an obvious gap was observed in the non-healed meniscus at 4 weeks (Figure 2). No significant intergroup differences were observed at 4, 8, and 12 weeks in the MHS (Table 1).

**Figure 3:**
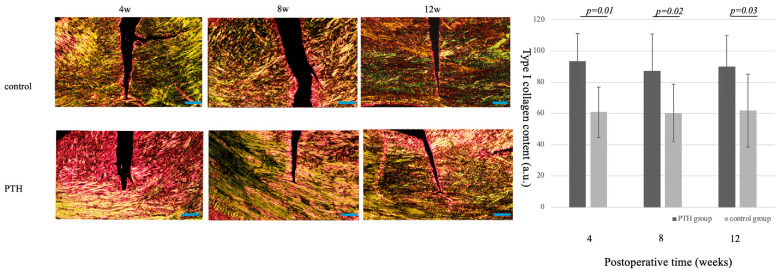
Picrosirius red staining of the meniscus of the control and PTH groups at 4, 8, and 12 weeks postoperatively. Scale bar = 200 μm. Quantitative analysis of type I collagen content was performed using picrosirius red staining. The type I collagen content in the outer two-thirds of the meniscal width was higher in the PTH group than in the control group at all time points. Data are presented as mean ± SD. *n* = 10 per group at each time point.

In the PTH group, yellow and red indicate collagen networks; in comparison, the control group exhibits greenish weak birefringence on Picrosirius red staining (Figure 3). Quantitative analysis showed that the type I collagen content was higher in the PTH group than that in the control group at all time points (4, 8, and 12 weeks) (*p* = 0.01, *p* = 0.02, and *p* = 0.03, respectively) (Figure 3).

**Figure 4:**
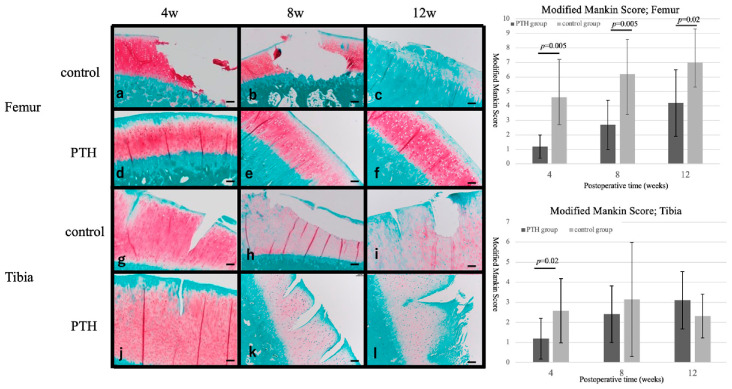
Representative images of chondral lesions of the femur and tibia and changes in their modified Mankin scores. Safranin O staining of the medial femur in the control ((**a**): 4 weeks, (**b**): 8 weeks, and (**c**): 12 weeks postoperatively) and PTH ((**d**): 4 weeks, (**e**): 8 weeks, and (**f**): 12 weeks postoperatively) groups. Safranin O staining of the tibial plateau in the control ((**g**): 4 weeks, (**h**): 8 weeks, and (**i**): 12 weeks postoperatively) and the PTH ((**j**): 4 weeks, (**k**): 8 weeks, and (**l**): 12 weeks postoperatively) groups. Control group (Femur): (**a**,**b**) show complete disorganization of the chondral structure. (**c**) shows pannus formation, surface irregularities, hypocellularity, and a severe reduction in matrix staining. The PTH group (Femur): (**d**,**f**) show a nearly normal cartilage structure. (**e**) shows slight reduction in matrix staining. The PTH group exhibited a significantly enhanced modified Mankin score than the control group at all time points. Control group (Tibia): (**g**) shows clefts in the calcified zone, hypocellularity, and a slight reduction in matrix staining. (**i**) shows clefts in the transitional zone, clusters of cells, and a severe reduction in matrix staining. The PTH group (Tibia) (**j**) shows clefts in the transitional zone and a moderate reduction. (**k**) shows clefts in the calcified zone and a severe reduction in matrix staining. (**l**) shows clefts in the transitional zone, hypocellularity, and a severe reduction in matrix staining. The PTH group exhibited a significantly better score than the control group at 4 weeks postoperatively. Scale bar = 100 μm. Data are presented as mean ± SD. *n* = 10 per each group at each time point.

Cartilage damage gradually progressed in the femur and tibia in both groups. In the PTH group, participants exhibited an improved modified Mankin score for the femur and tibia at 4 weeks compared to the control group (Femur: 1.2 ± 0.8 in the PTH group, 4.6 ± 2.7 in the control group, *p* < 0.05; Tibia: 3.8 ± 1.5 in the PTH group, 5.4 ± 1.9 in the control group, *p* < 0.05) (Figure 4).

## 3. Discussion

The most important finding of this study was that a single intra-articular injection of PTH (1–34) induced meniscal healing at the outer two-thirds of the meniscal width in a rabbit model of medial meniscus radial tear. The effect persisted for up to 8 weeks, accompanied by increased expression of type I collagen at the healing site, which increased the strength of the circumferential fibers.

Relatively few animal experiments on radial tears have been conducted to date. Pan et al. [2] reported that a bio-adhesive composed of silk fibroin incorporated with transforming growth factor β1 and crosslinked with phenylboronic acid–ionic liquid exhibited favorable healing of radial tears and reduced chondral damage in a rabbit model at 8 weeks postoperatively. The actual healing rate was not reported; nevertheless, complete healing of the white zone was not achieved. Mazy et al. [11] reported that, in a rat radial tear model, a single intra-articular injection of growth differentiation factor 5 increased meniscal healing at 6 weeks postoperatively. They evaluated meniscal healing in three regions, white–white, red–white, and red–red zones, and reported that healing was not observed in the white–white zone, even in the treated group. Rothrauff et al. [5] reported that performing meniscal repair augmented with photocrosslinkable methacrylate gelatin hydrogel containing transforming growth factor-β3 in a goat radial tear model improved meniscal healing and decreased osteoarthritic change in the knee joint. They reported that complete healing was achieved at the tear site in 75% of the specimens 24 weeks postoperatively. In our study, the PTH group exhibited an improved healing rate until postoperative week 8, although healing was not observed in the inner one-third of the meniscus, which is consistent with other reports. Although PTH (1–34) significantly improved the macroscopic healing rate, these improvements were not fully reflected in the MHS. This finding may be linked to the fact that the macroscopic healing rate primarily evaluates structural continuity of the meniscus, whereas the MHS additionally evaluates tissue maturity, including cellular morphology and matrix staining. Although reparative tissue frequently bridged the tear site, the regenerated tissue often lacked chondrocyte-like cells and Safranin O-positive matrix, suggesting that histological maturation remained incomplete.

Several theories have been proposed to explain the mechanisms underlying meniscal healing. Traditionally, vascularity has been reported to affect the healing of the injured meniscus [12]. In the clinical setting, meniscal repair concomitant with anterior cruciate ligament reconstruction demonstrated improved healing rates compared to isolated meniscus repair [13,14], which is attributed to growth factors released through bone tunnel creation [15]. In a parabiotic rat meniscal model, Terai et al. [16] reported that macrophages derived from circulating peripheral blood-borne cells accumulate in the regenerating meniscus during the early phase. The findings indicate the importance of vascularity in meniscal healing. They also reported that the number of macrophages derived from the synovial tissue was elevated in the latter phase of meniscal healing [16]. In a closely related mechanism, Nakagawa et al. [17] reported that synovial tissue is induced at the meniscal tear site, leading to tissue formation through the recruitment of synovial MSCs. Ozeki et al. [18,19] also reported that synovial MSCs attached to the injured site of the meniscus may transform into meniscal cells, leading to healing. In our study, healing was enhanced in the meniscal vascular zone following PTH (1–34). These findings may be related to enhanced repair responses in the vascularized region, increased collagen type I deposition, or local cell recruitment. However, the underlying cellular and molecular mechanisms were not investigated in our study. Further cellular and molecular studies are required to clarify the mechanisms underlying the effects of PTH (1–34) on meniscal healing.

PTH (1–34) has also been reportedly effective in the treatment of tendon injuries [19,20]. Yoon et al. [19] reported that systemic and local administration of PTH (1–34) resulted in increased Col I protein content and a higher ultimate tensile load at the repair site of a rat model of rotator cuff tears. Lee et al. [20] reported that in a murine flexor tendon injury model, systemic injection of PTH (1–34) resulted in increased expression of the *Col1a1* gene and higher tensile strength than PBS injection. Consistent with these findings, the PTH group demonstrated increased type I collagen deposition in the outer two-thirds of the meniscus. Although this increase was accompanied by greater circumferential fiber strength during the early postoperative period, the mechanical advantage was no longer evident at 12 weeks despite persistently higher type I collagen content. This finding suggests that increased collagen deposition alone may not fully determine the long-term mechanical properties of the repaired meniscus.

In most previous in vivo studies investigating the therapeutic effects of PTH (1–34), researchers have employed systemic administration, with doses generally ranging from 10 to 80 μg/kg [21]. In contrast, Chang et al. [22] suggested that intra-articular administration may achieve therapeutic efficacy at substantially lower doses than systemic administration because PTH (1–34) is delivered directly to the target tissue while minimizing systemic exposure. Furthermore, Nishino et al. [10] reported that in a rat model of meniscal defect, transplanted Achilles tendons treated with PTH (1–34) regenerated into meniscus-like tissue and caused reduced tibial cartilage damage until postoperative week 8. Furthermore, they reported that 3 μg/kg of PTH (1–34) did not induce ossification of the tissue; therefore, the same dose was used in our study.

In several studies, researchers have reported the effects of PTH (1–34) on meniscal defects. Zhao et al. [9] reported that in a canine model, transplantation of a 3D-printed poly scaffold seeded with BMSCs and PTH (1–34) after meniscectomy resulted in regenerated tissue similar to that of the native meniscus, and the knee joint exhibited less damage until postoperative week 12. Moreover, Nishino et al. reported increased type II collagen content in the meniscus generated from a PTH-injected tendon; however, we did not observe healing in the inner one-third of the meniscal width, a region rich in type II collagen in the native meniscus [23,24]. Intra-articular injections may have affected only the vascular zone of the meniscus, possibly resulting solely in an increase in type I collagen.

In our study, the PTH group exhibited a higher meniscal healing rate and less cartilage damage at 4, 8, and 12 weeks on the femoral side and at 4 weeks on the tibial side. The results of a previous study suggested that intra-articular PTH (1–34) exerts chondroprotective effects by preserving glycosaminoglycan content and suppressing chondrocyte apoptosis, thereby attenuating osteoarthritis progression. These findings indicate that, in addition to promoting meniscal healing, PTH (1–34) may contribute to preservation of the articular cartilage environment [21,22]. In addition, activation of PTH receptor 1 has been shown to stimulate chondrocyte proliferation and extracellular matrix production, including type II collagen, suggesting that PTH (1–34) also possesses chondroregenerative effects [25].

Although arthrofibrosis is characterized by excessive fibrotic tissue formation following joint injury or surgery, the potential effects of PTH (1–34) on sterile arthrofibrosis have not yet been fully elucidated and remain an important subject for future investigation.

This study has some limitations that must be acknowledged. First, radial tears of the anterior horn are uncommon in clinical practice. Second, the meniscal structure and healing capacity differ between rabbits and humans. Third, we did not conduct cellular or molecular analyses or an in vitro study to investigate the mechanisms underlying the effects of PTH (1–34) on meniscal healing. In addition, we did not perform immunohistochemical analyses using specific chondrocyte, vascular, and macrophage markers to further characterize the regenerated tissue. Therefore, the proposed mechanisms remain speculative and require further investigation.

## 4. Materials and Methods

### 4.1. Animals

All animal procedures were approved and conducted following the regulations of the Committee on Animal Research (Approval No. 23073, approved on 15 December 2023). Thirty skeletally mature female Japanese White rabbits aged 12–27 months (weight range, 3.3–4.3 kg; Japan SLC, Shizuoka, Japan) were used in this study, which was approved by our institution’s committee for animal experimentation. The rabbits were allocated to either the PTH group or the control group (*n* = 15 rabbits per group). Treatment allocation was not randomized. Five rabbits from each group were euthanized at 4, 8, and 12 weeks after surgery for subsequent macroscopic, biomechanical, and histological evaluations. The rabbits were housed in cages with free access to water and food in an air-conditioned environment. This study was conducted in accordance with ARRIVE 2.0 guidelines.

### 4.2. Surgical Procedure

**Figure 5:**
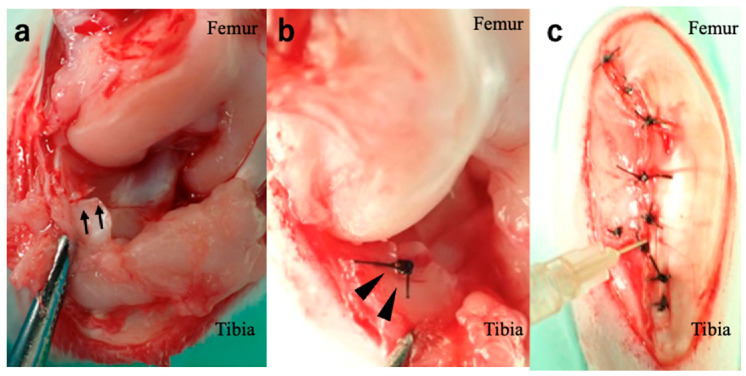
Surgical procedure. Left knee: (**a**) Radial tear at the anterior portion of the medial meniscus. The tear is indicated by an arrow. (**b**) A single suture was used to repair the tear. The arrowheads depict the repair sites. (**c**) After the closure of the joint capsule, 0.3 mL parathyroid hormone (1–34) or phosphate-buffered saline was injected.

The rabbits were anesthetized using subcutaneous injection of ketamine (50 mg/mL; Sankyo Co., Ltd., Tokyo, Japan) and xylazine (0.2 mg/mL; Bayer Co., Ltd., Tokyo, Japan) at a 10:3 ratio, administered at a dose of 1 mL/kg body weight. For each rabbit, the areas around both knee joints were shaved and cleaned using chlorhexidine gluconate solution. An orthopedic surgeon performed the surgical procedure (S.T.). A medial parapatellar incision was made at the knee joint, and the patella was dislocated laterally. A radial tear involving two-thirds of the meniscal width was created in the anterior portion of the medial meniscus after opening the joint capsule (Figure 5a). The tear was repaired with a single 5-0 nylon suture (Bear Medic Corp., Ibaraki, Japan) immediately after its creation (Figure 5b). The joint capsule was closed using 3-0 nylon (Bear Medic Corp., Ibaraki, Japan) suture. In the PTH group, PTH (1–34) (LKT Laboratories inc., Minnesota, USA) was injected intra-articularly into the knee joint (0.3 mL, 3 μg/kg), whereas the control group received PBS (Figure 5c).

After the operation, rabbits were housed in cages, and walking and hopping were restricted by cage size. The rabbits were sacrificed at 4, 8, and 12 weeks post-surgery. We excised the bilateral meniscus, femur, and tibia at the time of sacrifice. Five rabbits (10 knees) were included for each group at each time point.

### 4.3. Macroscopic Evaluation and Biomechanical Testing

**Figure 6:**
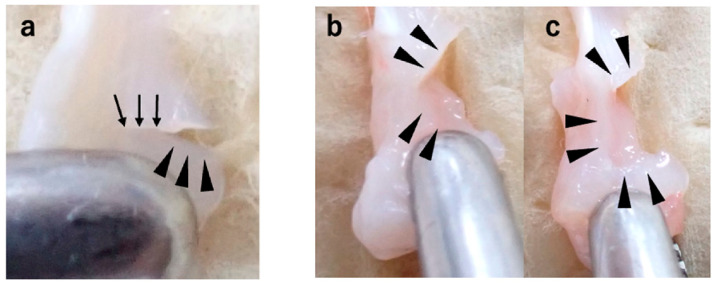
Macroscopical appearance of the medial meniscus. (**a**) Healed meniscus (left knee): half of the meniscal width is healed. (**b**) Non-healed meniscus (left knee): greater than two-thirds of the meniscal width is torn. (**c**) Non-healed meniscus (left knee): scar tissue is observed in the gap at the tear site. The healed site is depicted by the arrow, whereas the arrowhead depicts the non-healed site.

Immediately after the meniscal excision, the specimens were assessed macroscopically. Specimens in which the tear width was less than two-thirds of the meniscal width were considered healed (Figure 6a). Conversely, specimens in which two-thirds or more of the meniscal width was torn (Figure 6b), or in which a gap filled with scar tissue was present at the tear site (Figure 6c), were defined as non-healed (Figure 6a, healed; Figure 6b,c, non-healed).

After macroscopic evaluation, a single stitch with 1-0 blade silk (Bear Medic Corp., Ibaraki, Japan) was placed at the anterior and posterior meniscus segments. The upper and lower sutures were pulled with a 1N tensile load, and the displacement (widening distance) at the tear site was measured using ImageJ software (NIH, Bethesda, MD, USA). The analysis was performed using EZ Graph (Shimazu, Kyoto, Japan).

### 4.4. Histological Evaluation

After biomechanical testing, the entire medial meniscus was fixed in 4% paraformaldehyde solution for 24 h and embedded in paraffin blocks. Axial sections of 4 μm thickness were obtained and stained with hematoxylin–eosin, safranin O, and picrosirius red (Picro-Sirius Red Stain Kit; ScyTek Laboratories, Logan, UT, USA) following the manufacturer’s protocol [26]. An Olympus BX-511 microscope with a transmitted-light polarizer (U-POT; Olympus, Tokyo, Japan) and an analyzer (U-ANT; Olympus) as the second filter was used for evaluation. The image was rotated relative to the polarization plane, and a digital image was captured at the point of maximum brightness.

According to Junqueira et al. [26], fibers exhibiting strong birefringence with yellow-to-red coloration were identified as type I collagen; in comparison, type III collagen was identified as exhibiting weak birefringence with greenish coloration. Based on findings by Nakazawa et al. [27], the images were imported into ImageJ software version 1.54 r (National Institutes of Health, Bethesda, MD, USA) and 8-bit digitized. Non-collagenous fibrous tissue appeared black (0-bit), whereas a gray scale ranging from 1 to 255 bits represented collagenous fibrous tissue. Five areas (100 × 100 μm each) were randomly selected from the peripheral 2/3 of the meniscal width at the site of the radial tear and analyzed. The grayscale values were calculated as the averages of these regions, measured and documented following previously established methods.

Meniscus healing was evaluated using the meniscal healing score (MHS) [28,29], which evaluates the presence and quantity of extracellular matrix at the site of the meniscal tear. The scores ranged from 0 to 3 points: 0, no significant reaction; 1, reaction but no bridge connecting the two components; 2, connective tissue between the components; 3, explants with fibrous continuity between the two sides of the gap.

The femur and tibia were fixed in 4% paraformaldehyde solution for 24 h, decalcified in Morse’s solution for 3 weeks, and embedded in paraffin blocks. The specimens were cut and radially sectioned at 4 μm. The femur and tibia were stained with safranin O-fast green, and the modified Mankin score was used to evaluate cartilage damage. The histological evaluations were performed by two investigators. One investigator (S.T.) was a surgeon, and the other (K.I.) was an independent investigator blinded to the procedure. Evaluation was performed in a blinded manner.

### 4.5. Statistical Analysis

Pearson’s chi-square test was used to compare healing rates between the PTH and control groups. The Mann–Whitney U test was used to compare the widening distance and scores between the PTH and control groups. Kruskal–Wallis one-way analysis was used to compare scores across time points within each group. Interclass correlations and coefficients were determined by two orthopedic surgeons (S.T. and K.I), whereas intraclass correlation coefficients were determined by a single orthopedic surgeon (T.S.), separated by a 4-week interval. All analyses were performed using R (version 4.3.1) software. Statistical significance was set at *p* < 0.05.

## 5. Conclusions

A single injection of PTH (1–34) into the knee joint improved meniscal healing in a rabbit model of medial meniscus radial tear at 4 and 8 weeks, which was accompanied by increased type I collagen content at the healing site.

## Figures and Tables

**Table 1:** Meniscal Healing Score at 4, 8, and 12 weeks.

	4 Weeks	8 Weeks	12 Weeks	*p*-Value
Control	0.5 ± 0.8	0.4 ± 0.7	0.3 ± 0.7	0.84 **
PTH	1.6 ± 1.3	1.0 ± 1.2	0.8 ± 1.0	0.46 **
*p*-value	0.05 *	0.19 *	0.14 *	

* Mann–Whitney U test; ** Kruskal–Wallis one-way analysis.

## Data Availability

The original contributions presented in this study are included in the article Further inquiries can be directed to the corresponding author.

## Abbreviations

The following abbreviations are used in this manuscript:

BMSC	bone marrow mesenchymal stem cell
CI	confidence interval
MHS	meniscal healing score
MSC	mesenchymal stem cell
PBS	phosphate-buffered saline
PTH	parathyroid hormone
